# First Fall-Related Injuries Requiring Hospitalization Increase the Risk of Recurrent Injurious Falls: A Nationwide Cohort Study in Taiwan

**DOI:** 10.1371/journal.pone.0149887

**Published:** 2016-02-22

**Authors:** Carlos Lam, Jiunn-Horng Kang, Hsiao-Yu Lin, Hung-Chang Huang, Chia-Chieh Wu, Ping-Ling Chen

**Affiliations:** 1 Emergency Department, Department of Emergency and Critical Care Medicine, Wan Fang Hospital, Taipei Medical University, Taipei, Taiwan; 2 Graduate Institute of Injury Prevention and Control, College of Public Health and Nutrition, Taipei Medical University, Taipei, Taiwan; 3 Department of Emergency Medicine, College of Medicine, Taipei Medical University, Taipei, Taiwan; 4 Department of Physical Medicine and Rehabilitation, Taipei Medical University Hospital, Taipei Medical University, Taipei, Taiwan; 5 Department of Physical Medicine and Rehabilitation, School of Medicine, College of Medicine, Taipei Medical University, Taipei, Taiwan; 6 Department of Urology, Taipei Medical University Hospital, Taipei, Taiwan; 7 Division of Acute Care Surgery and Traumatology, Department of Surgery, Taipei Medical University Hospital, Taipei, Taiwan; University of Tuebingen, GERMANY

## Abstract

**Objectives:**

Recurrent falls not only have risk factors different from those of single falls but also have less favorable outcomes. The aim of our study was to determine the association between the injury characteristics of a first fall and the likelihood of recurrent injurious falls in a cohort of hospitalized patients.

**Methods:**

We designed a nationwide retrospective cohort study and selected hospitalized patients who had injurious falls between 2001 and 2010. Cox proportional hazards models were used to estimate the hazard ratios (HRs) of recurrent injurious falls requiring hospitalization in the following year on the basis of the patients’ demographic characteristics, comorbidities, and the characteristics of injuries from the first injurious fall requiring hospitalization.

**Results:**

Among the 504 512 patients hospitalized for injurious falls, 19 442 experienced recurrent injurious falls requiring hospitalization. The 1-year incidence of recurrent injurious falls requiring hospitalization was 3.85%. The incidence density was the highest within the 3-month period after the first injurious fall. The risk of recurrent injurious falls among patients aged 40 to 64, 65 to 74, and ≥ 75 years increased progressively (HR: 2.11, 95% confidence interval [CI]: 1.90–2.34; HR: 2.80, 95% CI: 2.51–3.11; and HR: 3.80, 95% CI: 3.42–4.23, respectively). The length of hospitalization (LOH) ≥ 15 or ≥ 31 days (HR: 1.39, 95% CI: 1.30–1.48; and HR: 1.59, 95% CI: 1.43–1.77, respectively) and injury to the head (HR: 1.59, 95% CI: 1.53–1.65) or spine (HR: 1.66, 95% CI: 1.59–1.74) were also found to be major risk factors.

**Conclusions:**

Our findings show that the LOH and head and spine injuries are associated with an increased risk of recurrent injurious falls leading to hospitalization. The risk of recurrent injurious falls requiring hospitalization increased significantly among adults older than 40 years. We suggest further research on the effects of injury characteristics associated with the first injurious fall requiring hospitalization and resultant anatomical damages on the risk of recurrent injurious falls requiring hospitalization. High-risk patients should receive tailored rehabilitation addressing their respective injuries within 3 months after hospital discharge.

## Introduction

Studies on recurrent falls rarely relate to the general population and have mostly focused on the older population or patients with specific diseases [1–5]. Among studies of community-dwelling older people, the Longitudinal Aging Study Amsterdam in the Netherlands reported a 1-year incidence rate of 11.5% for recurrent falls [2]. A study of community-dwelling older people in South Korea reported a 3-month recurrent fall rate of 11.2% [3]. In Taiwan and Japan, community studies of the older population have reported 1-year recurrent fall rates of 6.0% and 5.8%, respectively [4, 5]. However, a Japanese study including young adults reported a 3-year recurrent fall rate of 12.2% [6]. The discrepancy in recurrent fall rates among these studies is believed to be due to differences in the patient selection criteria, patient health status, and observation period.

Recurrent falls result in higher mortality and likelihood of admission to a nursing facility compared with single fall events [7,8], and have been reported to have different risk factors [4, 9]. A previous study indicated that recurrent falls are highly correlated with the physical, functional, and mental characteristics of a patient [10], and the risk factors include age, sex, physical decline, dizziness, chronic illness, drug use, and environmental conditions [9, 11–14]. Hence, to provide tailored intervention to prevent future recurrent injurious falls, emergency care providers should prioritize the identification of risk factors among patients who experienced an injurious fall [15].

Few published studies have explored the effects of the injury characteristics from first injurious falls on the risk of recurrent injurious falls [16]. Moreover, the studies that have examined these effects have not used a nationwide cohort of hospitalized patients with injurious falls including all age groups. Therefore, we designed a retrospective cohort study to determine the association between the injury characteristics of an injurious fall requiring hospitalization and the likelihood of recurrent injurious falls requiring hospitalization by using a nationwide population-based cohort of hospitalized patients. In addition, we used this cohort to calculate the incidence rate and incidence density of recurrent injurious falls requiring hospitalization within 1 year of the first injurious fall requiring hospitalization.

## Materials and Methods

### Data Source

Our study examined data from the National Health Insurance Research Database (NHIRD) provided by the Ministry of Health and Welfare (MHW). The National Health Insurance (NHI) program provides insurance coverage to more than 23 million enrollees, representing more than 99% of Taiwan’s total population [17]. The data regarding medications and treatment procedures are periodically reviewed by the NHI Administration, and the accuracy of the diagnoses, procedures, and prescriptions in the NHI claims data has been validated in previous studies [18–20]. We extracted data regarding inpatient expenditures by admissions, details of inpatient orders, ambulatory care expenditures by visits, the registry for beneficiaries, and household registration profiles from the complete claims data set recorded in the NHIRD between 2000 and 2011. To secure patient confidentiality, the MHW removed all identifiable patient information from the NHIRD. Our study was exempted from approval by the Institutional Review Board of Taipei Medical University (No: 201311009).

### Participant Selection

We selected only patients who were hospitalized for unintentional fall-related injuries (external codes E880-886 and E888). For the first injurious falls requiring hospitalization, we selected patients who had a first injurious fall requiring hospitalization within the 2001–2010 observation period. For the recurrent injurious falls requiring hospitalization, we selected patients who were hospitalized for injurious falls within 1 year after discharge from the first injurious fall requiring hospitalization. We excluded patients who were in a persistent vegetative state after the first fall (ICD-9-CM: 780.03) and patients who had a first injurious fall requiring hospitalization but whose data were incomplete regarding the discharge date.

### Measurements

A recurrent injurious fall requiring hospitalization was the outcome measure and was defined as the first hospitalization due to an injurious fall within 1 year after discharge from the hospitalization for the first injurious fall. One year was chosen as the observation period because most published studies have defined people who had recurrent falls as “persons who fell at least twice within 1 year” [13]. The cohort was divided into the following two groups: patients who experienced a recurrent injurious fall requiring hospitalization within the aforementioned observation period (recurrent injurious fall group), and patients who experienced only a single injurious fall requiring hospitalization (single injurious fall group).

Intensive care unit (ICU) stay, ventilator use, and length of hospitalization (LOH) following the first injurious fall were used to characterize the fall-related injuries. We divided the LOH into the following five groups: < 4 days, 4 to 7 days, 8 to 14 days, 15 to 30 days, and ≥ 31 days. This categorization is mainly based on the clinical practice patterns and experience of trauma surgeons in Taiwan, which have been highly influenced by the NHI. The injury locations were classified according to the Barell matrix: head, spine, torso, and extremity [21].

Demographic variables were sex, age, insurance premiums, enrollee category, marital status, and place of enrollment. A patient’s insurance premium was considered to represent the patient’s income level and socioeconomic status. The patients’ place of enrollment was divided into seven strata according to the categorization proposed by Liu et al. of Taiwan’s National Health Research Institutes [22], with Level 1 representing the most urbanized and Level 7 representing the least urbanized. This categorization has been widely used in studies in Taiwan because it provides the most accurate reflection of the real scenarios of urbanization. We used the urbanization variable as an indicator of the environmental factors affecting each patient [23, 24].

Comorbidities included various chronic illnesses related to recurrent falls [13, 25]. The analyzed comorbidities were cognitive impairment, depression, stroke, coronary artery disease, urinary incontinence, arthritis, dizziness, hypotension, diabetes mellitus, Parkinson disease, hypertension, cancer, asthma or chronic obstructive pulmonary disease, hyperlipidemia, and osteoporosis (S1 Appendix). We included only comorbidities diagnosed within the 1-year period before the hospitalization for the first injurious fall. We included only the disease which was diagnosed once on inpatient expenditures by admissions or twice on ambulatory care expenditures by visits. The comorbidity assessment was similar for each patient in this study.

### Statistical Analysis

First, the number of patients hospitalized for recurrent injurious falls was used as the numerator, and the total number of observed person-years was used as the denominator to calculate the incidence density. Subsequently, a univariate analysis was performed to compare the differences in the injury characteristics, demographic characteristics, and comorbidities between the recurrent injurious fall group and single injurious fall group. This was conducted using a Pearson chi-squared test for the categorical variables, a Cochran–Armitage trend test for the ordered categorical variables, and independent *t* tests for the continuous variables.

We used Cox proportional hazards models for multivariable analyses to evaluate the changes in variables of interest as different models were applied. All variables were divided into three categories: injury characteristics, comorbidity, and demographic characteristics. The injury characteristics were considered variables of interest, and comorbidity and demographic characteristics were considered confounding factors. Hazard ratios (HRs) and 95% confidence intervals (CIs) were calculated to estimate the risk of recurrent falls requiring hospitalization (outcome measure). Multiple Cox Regression analysis was then done among variables within each category. Only variables with significant differences were retained. Thus, the 3 categories of retained variables were sequentially entered according to this sequence: the variables of interest (injury characteristics) were entered first, and then the confounding factors including comorbidity and demographic characteristics were entered. Three Cox proportional hazards models were constructed: model 1, injury characteristics; model 2, model 1 and comorbidities; and model 3, model 2 and demographic characteristics.

We used a deviance test to compare the three models, and the best-fit model (*P* < .0001) was used to determine the risk of a recurrent injurious fall requiring hospitalization within the 1-year observation period after the first injurious fall requiring hospitalization. The statistical analysis was restricted to patients with no missing values in a specific variable. The patients who died within the 1-year observation period after discharge from hospitalization for the first injurious fall were considered censored. A two-sided *P* < .05 was considered to indicate a statistically significant difference. All of the statistical analyses were performed using the Statistical Analysis System (SAS) software, Version 9.3 (SAS Institute, Cary, NC, USA).

## Results

**Fig 1:**
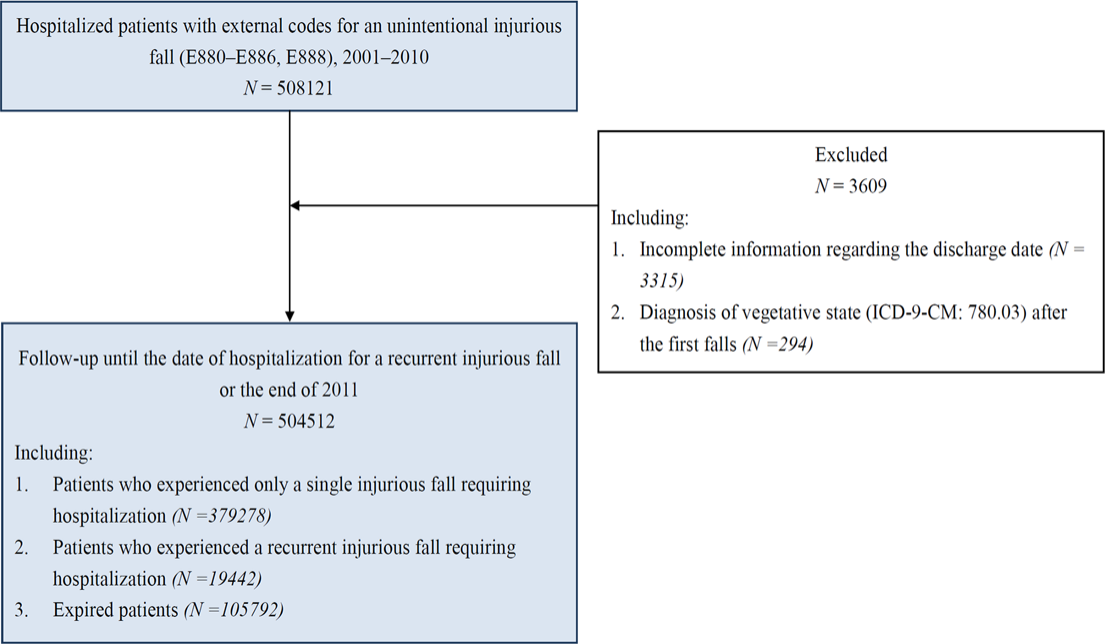
Patient selection from the National Health Insurance Research Database in Taiwan, 2001–2010.

**Fig 2:**
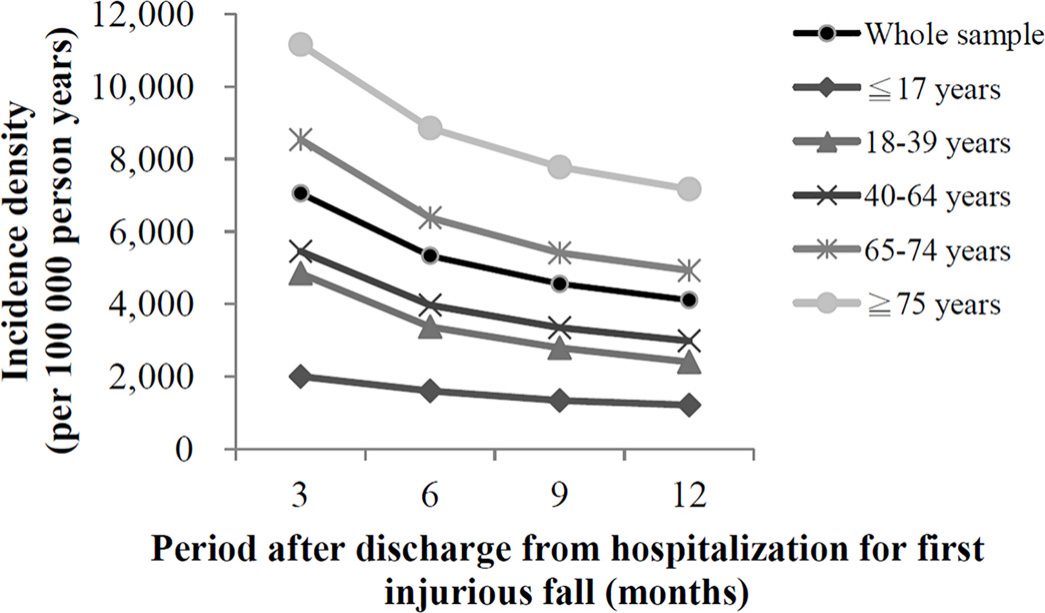
Incidence density of recurrent injurious falls requiring hospitalization in Taiwan, 2001–2010 (N = 504 512).

Our study examined 504 512 patients who were hospitalized for injurious falls between 2001 and 2010. Within the 1-year observation period, 19 442 patients experienced a recurrent injurious fall requiring hospitalization. A total of 105 792 patients were identified as expired during the aforementioned observation period (Fig 1). The 1-year incidence rate of recurrent injurious falls requiring hospitalization in our cohort was 3.85%. The incidence density of recurrent injurious falls requiring hospitalization was the highest within the 3-month period following discharge from hospitalization due to the first injurious fall (Fig 2). According to a univariate analysis, a significantly higher proportion of patients aged 65 years and older experienced a recurrent injurious fall requiring hospitalization compared with patients in the younger age groups (Table 1). The experience of a recurrent injurious fall requiring hospitalization was more common in patients with comorbidities; in addition, it was the more common in patients with hypotension than in those with other comorbidities (Table 2). We observed a positive trend between the LOH for the first injurious fall and the incidence of recurrent injurious falls. A higher percentage of patients with an LOH ≥ 15 days experienced a recurrent injurious fall requiring hospitalization (Table 3). The frequency of these falls was higher among patients with a spine or head injury (Table 3). Table 4 shows the results of the Cox proportional hazards modeling analyses. According to the results of the deviance test, Model 3 was the best-fit model for explaining the risk factors for recurrent injurious falls requiring hospitalization (*P* < .0001).

**Table 1:** Comparison of demographic variables between patients with and those without a recurrent injurious fall requiring hospitalization in Taiwan, 2001–2010.

Variable	Single injurious fall		Recurrent injurious fall		*P*
	(*N* = 485 070)		(*N* = 19 442)		
	*n*	%	*n*	%	
Sex					
Female	232 337	95.69	10 464	4.31	< .0001^a^
Male	249 163	96.54	8 940	3.46	
Missing^d^	3 570	0.74	38	0.20	
Mean age (SD) (years)	55.91	(24.67)	66.93	(20.24)	< .0001^b^
Age group (years)					
0–17	55 122	98.80	668	1.20	< .0001^c^
18–39	66 698	97.65	1608	2.35	
40–64	144 550	97.12	4290	2.88	
65–74	80 374	95.43	3851	4.57	
75+	134 755	93.75	8987	6.25	
Missing^d^	3571	0.74	38	0.20	
Enrollee category					
Self-insured	311 037	95.93	13 193	4.07	< .0001^a^
Dependent	172 947	96.53	6 222	3.47	
Missing^d^	1 086	0.22	27	0.14	
Insurance premiums (NTD/month)					
0–19 200	267 004	95.81	11 672	4.19	< .0001^c^
19 201–24 000	102 746	96.01	4 269	3.99	
24 001+	114 234	97.05	3 474	2.95	
Missing^d^	1 086	0.22	27	0.14	
Marital status					
Single	106 094	97.82	2 367	2.18	< .0001^a^
Married	246 023	96.18	9 784	3.82	
Divorced	27 847	95.81	1 217	4.19	
Widowed	97 979	94.25	5 976	5.75	
Missing^d^	7 127	1.47	98	0.50	
Place of enrollment					
1 (Most urbanized)	101 034	96.59	3 562	3.41	< .0001^c^
2	138 401	96.39	5 178	3.61	
3	81 231	96.26	3 160	3.74	
4	91 977	95.69	4 145	4.31	
5	15 462	95.21	778	4.79	
6	31 274	95.54	1 460	4.46	
7 (Least urbanized)	22 971	95.62	1 052	4.38	
Missing^d^	2 720	0.56	107	0.55	

SD, standard deviation; NTD, New Taiwan dollar

^a^ Pearson chi-squared test

^b^ *t* test

^c^ Cochran–Armitage trend test

^d^ Missing values in the table represent frequencies (column percentage)

**Table 2:** Comparison of comorbidities between patients with and those without a recurrent injurious fall requiring hospitalization in Taiwan, 2001–2010.

Variable		Single injurious fall		Recurrent injurious fall		*P* ^a^
		(*N* = 485 070)		(*N* = 19 442)		
		*n*	%	*n*	%	
Cognitive impairment	No	363 308	96.99	11 261	3.01	< .0001
	Yes	121 762	93.70	8 181	6.30	
Depression	No	465 669	96.29	17 924	3.71	< .0001
	Yes	19 401	92.74	1 518	7.26	
Stroke	No	438 204	96.43	16 226	3.57	< .0001
	Yes	46 866	93.58	3 216	6.42	
CAD	No	436 540	96.40	16 308	3.60	< .0001
	Yes	48 530	93.93	3 134	6.07	
Urinary incontinence	No	479 402	96.18	19 021	3.82	< .0001
	Yes	5 668	93.09	421	6.91	
Arthritis	No	423 813	96.44	15 648	3.56	< .0001
	Yes	61 257	94.17	3 794	5.83	
Dizziness	No	436 558	96.48	15 930	3.52	< .0001
	Yes	48 512	93.25	3 512	6.75	
Hypotension	No	483 923	96.16	19 323	3.84	< .0001
	Yes	1 147	90.60	119	9.40	
Diabetes	No	410 347	96.53	14 737	3.47	< .0001
	Yes	74 723	94.08	4 705	5.92	
Parkinson disease	No	473 127	96.26	18 407	3.74	< .0001
	Yes	11 943	92.02	1 035	7.98	
Hypertension	No	341 903	96.87	11 042	3.13	< .0001
	Yes	143 167	94.46	8 400	5.54	
Cancer	No	464 739	96.22	18 260	3.78	< .0001
	Yes	20 331	94.51	1 182	5.49	
COPD or asthma	No	449 942	96.32	17 210	3.68	< .0001
	Yes	35 128	94.03	2 232	5.97	
Hyperlipidemia	No	446 304	96.24	17 457	3.76	< .0001
	Yes	38 766	95.13	1 985	4.87	
Osteoporosis	No	459 772	96.33	17 531	3.67	< .0001
	Yes	25 298	92.98	1 911	7.02	

CAD, coronary artery disease; COPD, chronic obstructive pulmonary disease

^a^ Pearson chi-squared test

**Table 3:** Comparison of injury characteristics between patients with and those without a recurrent injurious fall requiring hospitalization in Taiwan, 2001–2010.

Variable		Single injurious fall		Recurrent injurious fall		*P*
		(*N* = 485 070)		(*N* = 19 442)		
		*n*	%	*n*	%	
ICU use	No	449 325	96.22	17 656	3.78	< .0001^a^
	Yes	35 745	95.24	1 786	4.76	
Ventilation use	No	470 004	96.17	18 702	3.83	< .0001^a^
	Yes	15 066	95.32	740	4.68	
LOH (days)						
≦3		176 275	97.04	5 372	2.96	< .0001^b^
4–7		171 794	96.17	6 838	3.83	
8–14		104 116	95.22	5 227	4.78	
15–30		26 172	94.36	1 564	5.64	
31+		6 713	93.84	441	6.16	
Injury location						
Extremity		301 560	96.82	9 902	3.18	< .0001^a^
Torso		26 614	95.53	1 245	4.47	
Head		109 818	95.31	5 403	4.69	
Spine		36 470	94.04	2 310	5.96	
Missing^c^		10 608	2.19	582	2.99	

ICU, intensive care unit; LOH, length of hospitalization

^a^ Pearson chi-squared test

^b^ Cochran–Armitage trend test

^c^ Missing values in the table represent frequencies (column percentage).

**Table 4:** Risk factors for a recurrent injurious fall requiring hospitalization in Taiwan, 2001–2010, according to Cox proportional hazards models.

Variable	Model 1		Model 2		Model 3	
	HR	95% CI	HR	95% CI	HR	95% CI
Injury location of first fall						
Extremity	1.00		1.00		1.00	
Torso	1.43	(1.35, 1.52)	1.44	(1.35, 1.53)	1.47	(1.38, 1.56)
Head	1.60	(1.54, 1.66)	1.54	(1.49, 1.60)	1.59	(1.53, 1.65)
Spine	1.83	(1.74, 1.91)	1.68	(1.60, 1.76)	1.66	(1.59, 1.74)
Other variables of injury characteristics						
ICU use	0.89	(0.83, 0.95)	0.93	(0.87, 0.99)	0.93	(0.87, 0.99)
Ventilation use	0.88	(0.80, 0.97)	0.90	(0.82, 0.99)	0.89	(0.81, 0.97)
LOH (days)						
≦3	1.00		1.00		1.00	
4–7	1.35	(1.30, 1.39)	1.23	(1.19, 1.28)	1.12	(1.08, 1.17)
8–14	1.79	(1.72, 1.86)	1.47	(1.41, 1.53)	1.24	(1.19, 1.29)
15–30	2.04	(1.92, 2.17)	1.61	(1.51, 1.71)	1.39	(1.30, 1.48)
31+	2.29	(2.05, 2.55)	1.78	(1.60, 1.99)	1.59	(1.43, 1.77)
Comorbidity						
Cognitive impairment			1.51	(1.45, 1.57)	1.36	(1.31, 1.41)
Depression			1.36	(1.29, 1.44)	1.42	(1.35, 1.50)
History of stroke			1.09	(1.05, 1.14)	1.04	(1.00, 1.09)
CAD			1.13	(1.08, 1.18)	1.04	(1.00, 1.09)
Urinary incontinence			1.21	(1.09, 1.33)	1.14	(1.03, 1.25)
Arthritis			1.15	(1.11, 1.20)	1.06	(1.02, 1.10)
Dizziness			1.11	(1.06, 1.16)	1.05	(1.00, 1.10)
Hypotension			1.47	(1.22, 1.77)	1.46	(1.21, 1.76)
Diabetes			1.32	(1.28, 1.37)	1.29	(1.24, 1.33)
Parkinson disease			1.40	(1.31, 1.49)	1.26	(1.18, 1.35)
Hypertension			1.23	(1.19, 1.27)	1.02	(0.99, 1.06)
Cancer			1.24	(1.16, 1.31)	1.14	(1.08, 1.22)
COPD or asthma			1.24	(1.18, 1.29)	1.12	(1.07, 1.17)
Hyperlipidemia			0.90	(0.85, 0.94)	0.94	(0.89, 0.99)
Osteoporosis			1.40	(1.33, 1.47)	1.28	(1.22, 1.35)
Demographics						
Sex						
Female					1.00	
Male					1.04	(1.00, 1.07)
Age group (years)						
0–17					1.00	
18–39					1.80	(1.63, 2.00)
40–64					2.11	(1.90, 2.34)
65–74					2.80	(2.51, 3.11)
75+					3.80	(3.42, 4.23)
Enrollee category						
Self-insured					1.00	
Dependent					1.05	(1.01, 1.09)
Insurance premiums (NTD/month)						
0–19 200					1.00	
19 201–24 000					0.95	(0.92, 0.99)
24 001+					0.84	(0.81, 0.88)
Marital status						
Single					1.00	
Married					0.93	(0.88, 0.99)
Divorced					1.21	(1.12, 1.31)
Widowed					1.07	(1.00, 1.14)
Place of enrollment						
1					1.00	
2					1.06	(1.01, 1.11)
3					1.11	(1.05, 1.16)
4					1.17	(1.12, 1.23)
5					1.18	(1.09, 1.28)
6					1.14	(1.07, 1.22)
7					1.17	(1.09, 1.26)
Model fit statistics						
-2 Log likelihood	490 471.84		486 739.66		478 909.83	
Model compared	1 vs null^a^		2 vs 1		3 vs 2	
Deviance	1092.42		3732.18		7829.83	
ΔDF	6		15		17	
*P*	< .0001		< .0001		< .0001	

NTD, New Taiwan dollar; CAD, coronary artery disease; COPD, chronic obstructive pulmonary disease; ICU, intensive care unit; ΔDF, difference between degrees of freedom; LOH, length of hospitalization; HR, hazard ratio; CI, confidence interval

^a^ Model with no covariates, -2 Log likelihood = 492 626.74

Regarding the injury location of the first injurious fall requiring hospitalization, the risk of recurrent injurious falls requiring hospitalization was significantly increased among patients with head or spine injuries (HR: 1.59, 95% CI: 1.53–1.65; and HR: 1.66, 95% CI: 1.59–1.74, respectively). The risk of these falls was significantly increased among patients with an LOH ≥ 15 or ≥ 31 days (HR: 1.39, 95% CI: 1.30–1.48; and HR: 1.59, 95% CI: 1.43–1.77, respectively). Among the comorbidities, hypotension was associated with the highest risk (HR: 1.46, 95% CI: 1.21–1.76). Cognitive impairment (HR: 1.36, 95% CI: 1.31–1.41), depression (HR: 1.42, 95% CI: 1.35–1.50), diabetes (HR: 1.29, 95% CI: 1.24–1.33), Parkinson disease (HR: 1.26, 95% CI: 1.18–1.35), and osteoporosis (HR: 1.28, 95% CI: 1.22–1.35) were also identified as significant risk factors. Model 3 revealed a positive trend between age and a recurrent injurious fall requiring hospitalization. The highest risk was found among patients aged 75 years and older (HR: 3.80, 95% CI: 3.42–4.23), and the risk among the ages of 40 to 64 and 65 to 74 years was also significantly higher (HR: 2.11, 95% CI: 1.90–2.34; and HR: 2.80, 95% CI: 2.51–3.11, respectively) compared with that in the younger age groups. A lower risk was found among the patients with higher insurance premiums (HR: 0.84, 95% CI: 0.81–0.88).

## Discussion

On the basis of a nationwide population-based cohort, our study indicated that the 1-year incidence rate of a recurrent injurious fall requiring hospitalization was 3.85%. We used the total number of patients hospitalized for fall-related injuries as the denominator to calculate the incidence of recurrent injurious falls requiring hospitalization. Most studies on recurrent falls have surveyed only the inhabitants of specific communities and calculated the incidence by using the community population as the denominator [2–6]. Because our study included only patients who were hospitalized for injurious falls, we expected the incidence of recurrent injurious falls requiring hospitalization to be lower compared with that in other studies that included any fall events with or without injury or hospitalization. Including patients from all age groups may also have contributed to a lower incidence in our study because younger people are healthier and are less likely to be admitted to the hospital for fall-related injuries. The inconsistencies in the recurrence of falls among different investigations are attributable to the diverse locations of each survey [26], observation times [12], methods of collecting information [4], definitions of sampling [27], and characteristics of patients such as age and health status [28]. Such variabilities in study design limit the ability to directly compare findings.

Furthermore, we determined that the incidence density ratio of recurrent injurious falls requiring hospitalization for the 3-month and 1-year follow-up periods was 1.72 and found that the incidence density was higher in the older population by using a time-trend analysis stratified by age group (Fig 2). Hence, the advantages of providing interventions to prevent recurrent injurious falls requiring hospitalization during the first 3 months after hospitalization for the first injurious fall should be emphasized [29].

Although previous studies have emphasized that experiencing a fall is a major risk factor for recurrent falls [4, 12, 30, 31], the effect of the injury characteristics of the first injurious fall on the risk of recurrent injurious falls has not been thoroughly investigated. Spector et al. reported that severe injuries, spinal cord injury, and traumatic brain injury were associated with increased hospital readmission rates and that markers of injury severity, including the LOH, were associated with the readmission rate [32]. Newgard et al. reported that the LOH is a valid indicator of injury severity, especially for severe injuries [33]. After we adjusted for the demographic characteristics and comorbidities, the best-fit model indicated that the LOH for the first injurious fall, a proxy of injury severity, was associated with a significant risk of recurrent injurious falls requiring hospitalization.

In addition, the best-fit model showed that patients with fall-related injuries to the head or spine requiring hospitalization were associated with an increased risk of a recurrent injurious fall requiring hospitalization. Results suggested that injury to the central nervous system, which causes impairments to cognition, coordination, and balance, might contribute to an increased risk of recurrent injurious falls requiring hospitalization.

Our study suggests that the injury characteristics associated with the first injurious fall requiring hospitalization, such as the LOH and head or spine injury, are risk factors for recurrent injurious falls requiring hospitalization. We emphasize that future research should focus not only on the injury characteristics of first injurious falls but also on the resultant anatomical damage, because such damage increases the risk of recurrent injurious falls. Future research should aim to provide individually tailored rehabilitation programs that address the specific injury characteristics (e.g., severity and locations) and resultant anatomical damage of patients hospitalized for fall-related injuries [34, 35].

The best-fit model indicated that the risk of a recurrent injurious fall requiring hospitalization was higher among patients with hypotension than among patients with other types of comorbidity. Hypotension prevents the brain from receiving sufficient blood, causing syncope and dizziness; this effect is exacerbated in patients who have dehydration, autonomic dysfunction, or polypharmacy [36]. Hypotension was reported as a modifiable risk factor for recurrent falls. Hence, screening for hypotension in patients hospitalized for fall-related injuries should not be underestimated [37].

In the best-fit model, we observed that age was a significant risk factor for recurrent injurious falls requiring hospitalization. The risk of these falls increased significantly among adults older than 40 years. Physical weakness, social withdrawal, functional decline, and polypharmacy have been widely reported to be associated with an increased incidence of recurrent falls among senile patients [4, 11, 13]. Talbot et al. conducted a comparative study on falling among different age groups; 21% of middle-aged adults reported falling at least once in a 2-year period [38]. Chronic diseases, increased use of medication, and a reduced level of physical activity may explain such results. Although occupational injury was reported as the most common cause of falling among middle-aged adults in Taiwan [39], the etiology and injury characteristics of recurrent injurious falls may be different. Hence, with consideration of societal productivity and contribution, preventing recurrent injurious falls in middle-aged people is crucial.

### Strength and Limitations

Our study used a nationwide cohort of hospitalized patients to avoid the common shortcomings of community-based surveys, such as small sample sizes, specific age groups, and recall bias [30, 40]. Using this cohort enabled us to accurately calculate the incidence rate and incidence density of recurrent injurious falls requiring hospitalization as well as assess the various risk factors for these falls, including injury severity and location, among the entire population.

The NHIRD does not include data on lifestyle and environmental factors. Therefore, we used surrogate measures for these variables. The results of physical, psychological, and laboratory examinations are also not recorded in the NHIRD. These insufficiencies limit us in exploring further the relationship between diseases and the risk of recurrent injurious falls. We cannot obtain information on falls that occurred during hospitalization from this data set. For patients who moved abroad during the 1-year observation period, we cannot determine the exact date of the move because the NHIRD data are deidentified. Therefore, we can only consider the possibility that patients moved abroad as a limitation. On the basis of our analyses, the patients with a high risk of recurrent falls were mostly older than 65 years. We believe that patients in this age group are unlikely to emigrate abroad. Therefore, the bias caused by this limitation should be small. Because the NHI is mandatory in Taiwan and covers more than 99% of the total population, the possibility for Taiwanese residents to withdraw from the NHI should be small and the influence can be neglected.

Limiting participant selection to hospitalized patients excluded recurrent falls that resulted in death before hospital admission or minor injuries for which medical attention or hospitalization was not necessary. Thus, the results of our study cannot represent all recurrent injurious falls. In addition, excluding patients with incomplete information regarding the discharge date may have led to selection bias, although the effect should be minimal.

## Conclusions

We found that recurrent injurious falls requiring hospitalization occur most frequently within 3 months after hospitalization for a first injurious fall, and that the injury characteristics associated with the first injurious fall influence the risk of recurrent injurious falls requiring hospitalization; specifically, a longer LOH and head or spine injury are correlated with a higher risk.

Our study suggests that the risk factors for recurrent injurious falls among adults older than 40 years who were hospitalized for fall-related injuries must be evaluated further. We also suggest further research on the effects of injury characteristics associated with the first injurious fall requiring hospitalization and resultant anatomical damages on the risk of recurrent injurious falls requiring hospitalization. Finally, we recommend that patients hospitalized for injurious falls who are at a high risk of recurrent injurious falls requiring hospitalization receive early interventions within 3 months after hospital discharge. Such interventions should include individually tailored rehabilitation that addresses the patients’ particular injury characteristics.

## Supporting Information

S1 Appendix ICD-9-CM codes for comorbidities. (DOCX)
