# Supplementary material for: First Fall-Related Injuries Requiring Hospitalization Increase the Risk of Recurrent Injurious Falls: A Nationwide Cohort Study in Taiwan
Source: PLoS One. 2016 Feb 22;11(2):e0149887. doi: 10.1371/journal.pone.0149887 (PMC4763332; doi:10.1371/journal.pone.0149887)
Supplement: S1 Appendix — (DOCX) [file pone.0149887.s001.docx]

Appendix. ICD-9-CM codes for comorbidities

| Comorbid conditions | ICD-9-CM |
| --- | --- |
| Cognitive impairment | 290, 292.81, 293, 294.1, 294.9, 310.1, 331.0–331.2, 331.7–331.9, 348, 349, 437.0–437.1, 438.0, 780, 780.02, 780.93, 780.97, 797 |
| Depression | 296.2, 296.3, 300.4, 301.12, 309.0, 309.1, 311 |
| Stroke | 430–436, 437.1, 437.3, 437.6–437.9, 438 |
| Coronary artery disease | 410–414 |
| Urinary incontinence | 596.51, 625.6, 788.31–788.33,788.36, 788.4 |
| Arthritis | 714.0–714.2, 715 |
| Dizziness | 780.4 |
| Hypotension | 458 |
| Diabetes mellitus | 250 |
| Parkinson disease | 332, 333.0 |
| Hypertension | 401–404, 437.2 |
| Cancer | 140–208, 230–239 |
| Asthma or chronic obstructive pulmonary disease | 491.2, 493, 496, 506.4 |
| Hyperlipidemia | 272.0–272.5, 272.7–272.9, 374.51 |
| Osteoporosis | 733 |
